# Retraction: Salame et al. Is Increasing Age Associated with Higher Rates of Intercostal Arteries Vulnerable to Laceration? A Point of Care Ultrasound Study. *J. Clin. Med.* 2022, *11*, 5788

**DOI:** 10.3390/jcm12082905

**Published:** 2023-04-17

**Authors:** Gerard Salame, Elizabeth Wittrock, Hardik Patel, Brant Hafen, Ayal Levi, Tyler Millard

**Affiliations:** Saint Joseph Hospital, Denver, CO 80218, USA

The journal retracts the article, ‘Is Increasing Age Associated with Higher Rates of Intercostal Arteries Vulnerable to Laceration? A Point of Care Ultrasound Study’ [1] due to lack of an appropriate Institutional Review Board approval. 

Following publication, the authors contacted the editorial office with concerns related to the accuracy and validity of the Institutional Review Board Statement published in their article [1]. The authors informed the journal that an issue occurred during the initial approval process and that the approval was administratively withdrawn. 

Adhering to our complaints policy, an investigation was conducted that confirmed that the study did not have a valid Institutional Review Board approval. The Institutional Review Board investigated the matter and requested that the article be withdrawn. The article is therefore retracted. 

This retraction was approved by the Editor in Chief of the *Journal of Clinical Medicine*.

The authors agreed to this retraction.

## References

[1] Salame G., Wittrock E., Patel H., Hafen B., Levi A., Millard T. (2022). Is Increasing Age Associated with Higher Rates of Intercostal Arteries Vulnerable to Laceration? A Point of Care Ultrasound Study. J. Clin. Med..

